# Interactive robots for health in Europe: Technology readiness and adoption potential

**DOI:** 10.3389/fpubh.2023.979225

**Published:** 2023-03-13

**Authors:** Britt Östlund, Monica Malvezzi, Susanne Frennert, Michael Funk, Jose Gonzalez-Vargas, Kilian Baur, Dimitris Alimisis, Freygardur Thorsteinsson, Antonio Alonso-Cepeda, Guillaume Fau, Florian Haufe, Massimo Di Pardo, Juan C. Moreno

**Affiliations:** ^1^Department of Biomedical Engineering and Health Systems, Royal Institute of Technology (KTH), Stockholm, Sweden; ^2^Department of Information Engineering and Mathematics, University of Siena, Siena, Italy; ^3^Internet of Things and People Research Center, Malmö University, Malmö, Sweden; ^4^Cooperative Systems, Faculty of Computer Science, University of Vienna, Vienna, Austria; ^5^Ottobock SE & Co., KGaA (OBG), Duderstadt, Germany; ^6^CYBATHLON, ETH Zürich, Zürich, Switzerland; ^7^European Lab for Educational Technology (EDUMOTIVA), Athens, Greece; ^8^ÖSSUR, Reykjavik, Iceland; ^9^Department of Technological Innovation/ACCIONA Construction, Madrid, Spain; ^10^Space Applications Services (SA), Brussels, Belgium; ^11^Sensory-Motor Systems Lab, Institute of Robotics and Intelligent Systems, ETH Zürich, Zürich, Switzerland; ^12^SPW, Research and Innovation Department, Centro Ricerche Fiat (CRF), Orbassano, Italy; ^13^Neural Rehabilitation Group, Cajal Institute, Spanish National Research Council (CSIC), Madrid, Spain

**Keywords:** social robots, interactive robots, healthcare robots, educational robots, technology readiness

## Abstract

**Introduction:**

Social robots are accompanied by high expectations of what they can bring to society and in the healthcare sector. So far, promising assumptions have been presented about how and where social robots are most relevant. We know that the industry has used robots for a long time, but what about social uptake outside industry, specifically, in the healthcare sector? This study discusses what trends are discernible, to better understand the gap between technology readiness and adoption of interactive robots in the welfare and health sectors in Europe.

**Methods:**

An assessment of interactive robot applications at the upper levels of the Technology Readiness Level scale is combined with an assessment of adoption potential based on Rogers' theory of diffusion of innovation. Most robot solutions are dedicated to individual rehabilitation or frailty and stress. Fewer solutions are developed for managing welfare services or public healthcare.

**Results:**

The results show that while robots are ready from the technological point of view, most of the applications had a low score for demand according to the stakeholders.

**Discussion:**

To enhance social uptake, a more initiated discussion, and more studies on the connections between technology readiness and adoption and use are suggested. Applications being available to users does not mean they have an advantage over previous solutions. Acceptance of robots is also heavily dependent on the impact of regulations as part of the welfare and healthcare sectors in Europe.

## 1. Introduction

This paper investigates the social uptake of interactive robots in the healthcare sector in Europe. More specifically, rather than discussing about their usefulness for certain target groups, the paper seeks to answer the questions on what is available, and why some robots are not adopted despite high technical maturity, i.e., those that are already implemented or are about to be implemented in a social context. In this paper, the term “interactive robots” refers to robots close to individual users and which work in direct interaction with the individual. The purpose of the study is, firstly, to assess the relationship between technology readiness and adoption with respect to several products in the health sector, using the Technology Readiness Level (TRL) scale, which traces the path of innovation from ideas, tests, and demonstrations of prototypes to commercialization and procurements (1). Secondly, an assessment of adoption potential is made based on Rogers' theory of diffusion of innovation (2): this theory assumes that just because an application is deemed technology ready valued by manufacturers or researchers, it does not mean that it will be adopted by users.

The goal of the analysis is to understand how we can increase the social uptake of robots and what prevents this. We have come a long way in developing robots, but where are we in terms of implementing them in “real life” outside of research laboratories and factories? What is missing that prevents products with high technology readiness from being adopted? And where in the health sector are interactive robots already being used?

The evaluation we present in this work, being made by stakeholders, rather than end users, gives a well-founded assessment of solutions that have reached the level of robot applications already implemented or about to be implemented. This paper does not seek to answer questions about the contextualization of robots in real life, but rather presents the perspectives of researchers and companies that provide robot applications which have reached the stage of being full commercial applications, i.e., technology available for consumers. Thus, the current study constitutes a step between pure speculation and studies into how robots are used in real life.

The study is part of the European Commission-funded Horizon 2020 project Inclusive robotics for a better society—INBOTS, and more specifically, seeks to promote uptake of robotics in the field of health. Data collection was carried out by stakeholders representing 14 European universities and research laboratories, six businesses and five facilitators (Table 1).

**Table 1:** List of the stakeholders that collaborated in collecting data.

**Universities and research labs**
Scuola Superiore de Studi Universitari e di Perfezionamento Sant'Anna (SSSA)
Complutense University of Madrid (UCM)
University of Twente (UT)
Vrije Universiteit Brussel (VUB)
Swiss Federal Institute of Technology in Zurich (ETH)
Università Degli Studi di Siena (UNISI)
Dublin City University (DCU)
University of Leeds (UNIVLEEDS)
University of Vienna (UNIVIE)
Universiteit Utrecht (UU)
City University in London (CITY)
Royal Institute of Technology in Stockholm (KTH)
European Lab for educational technology (EDUMOTIVA)
Agencia Estatal Consejo Superior de Investigaciones Científicas (CSIC)
**Businesses**
Össur (ÖSSUR)
Otto Bock Healthcare Products GmbH (OBHP)
Centro Ricerche Fiat (CRF)
ACCIONA Construcción (ACC)
Space Applications Services NV (SAS)
Pal Robotics (PAL)
**Consultants and authorities (facilitators)**
Fundación Tecnalia Research & Innovation (TECNALIA) ES
Deutsches Institut für Normung (DIN) DE
VDI/VDE-IT Innovation + Technik GmbH (VDI/VDE-IT) DE
PKF ATTEST INNCOME (INNCOME) ES
IUVO S.r.l. (IUVO) IT

INBOTS (3) project aimed to coordinate and support relevant efforts in the field that covers those robots that are in close proximity and interact with a person, the main goal of the project was to create a community hub bringing together experts from different and complementary fields to debate and create a responsible research and innovation paradigm for Interactive Robotics and therefore to provide a platform establishing a working synergy between the main pillars covering the main stakeholders.

Robotic technologies are progressing fast, continuously delivering new and powerful technologies creating new opportunities for people and potentially able to transform the society in the near future. However, this evolution will also create new dangers and responsibilities that need to be elucidated and contained. Furthermore, the rapid advances in robotics may be difficult to understand for the public, with negative perceptions and overestimated expectations that should be clarified. Beyond the research presented in this paper, all the stakeholders involved in the project were therefore invited to collaborate to identify the most important aspects needed for an effective and responsible research and innovation in robotics. The INBOTS structure covered the following pillars: the technical expertise pillar, the business expertise pillar, the ethical, legal, and socio-economic (ELSE) expertise pillar, as well as the end-users, policy makers and general public pillar.

A first investigation presented to the European Commission in 2019 highlighted that compared to industry, transport, and logistics, where robots have been used for a long time to streamline production and improve the work environment, there was only fragmented knowledge about the use of robots in healthcare (4). Although some robots had been established with great success in microsurgery and assisting people with disabilities, to the best of our knowledge, comprehensive overviews and discussions of the health sector in a broad sense are still missing. Even in fields, such as education and implementation of interactive robots in public places, there is a lack of knowledge about what works and what does not.

The discussion about robots likely to be adopted, thus far has been mainly focused on commercial trends, investigating in particular on what is required for robots to function in human contexts and the challenges associated with the introduction of specific kinds of robots (5–7). In particular, the review presented in Royakkers and van Est (6) discusses the societal issues raised by the new robotics: which robot technologies are coming, their capabilities and potentialities, but also the ethical and regulatory questions they will raise. Specifically for the healthcare sector, (5) investigates the users' point of view (care staff and potential care receivers), on their assumptions, expectations and understandings, while (7) focuses on the impact of impact of robots in multiple medical domains.

Some evaluations shows that there is a lack of knowledge about the context and ecosystems in which robots are supposed to be implemented (8). Predictions are especially difficult to make in areas where robots have not been used before (9). A clear trend is that robots that have long been used in manufacturing on assembly lines are now being discussed as consumer products and as part of providing care and services to older adults at home (10, 11).

Robots can mean many different things. Today, there are a variety of applications designed for different purposes. Relevant organizations, such as the Institute of Electrical and Electronics Engineers (IEEE)^1^ and the Robotic Industries Association (RIA), have come up with their own classifications to provide at least some loose structure when addressing the extremely broad field of robotics. The RIA defines service robots as a new category of robots for use outside manufacturing, such as in agriculture, logistics, cleaning, medicine, customer service, hospitality, and personal assistance.

Thus, even this definition shows that robotics is a field undergoing strong development that renders all definitions provisional. However, this should not prevent us from assessing upcoming robot applications. Hence, the purpose of this paper is not to make conjectures about the future uses of robots, but rather to evaluate the current uptake of robots in the health sector, specifically in Europe, in terms of their adoption potential. Evaluations were made considering a set of robots entering the higher levels of the Technology Readiness Level (TRL) scale from 2015 up to today. This includes technologies tested in the intended environment, pre-commercialized or fully commercialized according to the TRL scale issued by the European Commission as part of the Horizon 2020 program (1) (Table 2). In this paper, with the term “health” we refer to any activities concerned with physiological or cognitive health, including wellness, such as enjoyment from participating in sports (12), educational activities (13), and biomedical perspectives (14).

**Table 2:** The technology readiness levels scale (TRL).

TRL 0	Idea. Unproven concept, no testing has been performed
TRL 1	Basic research
TRL 2	Technology formulation. Concept and application have been formulated
TRL 3	Applied research. First laboratory tests completed
TRL 4	Small scale prototype built in a laboratory environment
TRL 5	Large scale prototype tested in intended environment
TRL 6	Prototype system tested in intended environment
TRL 7	Pre-commercial demonstration system
TRL 8	Commercial system launched
TRL 9	Full commercial application, technology available for consumers

Looking at the available statistics on the social uptake and acceptance of robots in Europe, these do not provide much support for catching up with developments beyond industrial robots and transportation. Eurostat, the statistical office of the European Union, provides a clear picture of developments in the industry in terms of both products and services, but lacks the corresponding indicators for uptake and acceptance among citizens (15). In the EU, 25% of large enterprises use robots, along with 12% of medium sized enterprises (employing 50–249 persons) and 5% of small enterprises (employing 10–49 persons). Enterprises more commonly use industrial robots than service robots. Enterprises use service robots mainly for warehouse management systems (44%), followed by transportation of people and goods (22%), cleaning or waste disposal tasks, and assembly works (21% each).

In the absence of statistics on the social uptake of robots among the public, it can be fruitful to study the statistics available on Internet use and share the long-established notion that technological development is evolutionary (16). One of the most well-established theories about what contributes to people adopting new technology is Rogers' theory of diffusion of innovation built on familiarity (2). In this work, the author presents five factors that help to explain what makes users feel safe enough to accept new technologies:

- Relative advantage, defined as the “degree to which an innovation is perceived as being better than the idea it supersedes.”
- Compatibility, defined as the “degree to which an innovation is perceived as consistent with the existing values, past experiences, and needs of potential adopters.”
- Complexity, defined as the “degree to which an innovation is perceived as relatively difficult to understand and use.”
- Trialability, defined as the “degree to which the innovation may be tried and modified.”
- Observability, defined as the “degree to which the results of the innovation are visible to others.”

Rogers' theory was used in this study to design of a tool for evaluating the adoption potential of a selection of interactive robots that can be adopted in the field of health.

With Rogers' theory in mind, it can be assumed that high rates of Internet use among Europeans may help pave the way for the adoption of robotics as this habit may make the user less insecure about trying new similar technologies. The adoption rate of IT is both high and encompasses key areas of society. In 2021, 92% of European citizens access the Internet every day (15). The figures range from 84% in Bulgaria, to 99% in Luxemburg and Nederlands. Citizens who never use the Internet in 2021 amount to 8% in total.

In 2016, 85% of European households had access to the Internet, compared to 70% in 2010, while ten years earlier this kind of statistic was barely developed at European level (17). There are differences between age groups. For example, smartphone access per given age group was as follows: 16–24 years, 94%; 25–34 years, 91%; and 65–74 years, 48%.

However, the adoption of IT alone does not guarantee the adoption of robots. According to Rogers' theory, IT use helps to reduce the uncertainty of the individual when encountering new similar applications, but the opportunity and willingness to take on new technology is also affected by accessibility, regulations, and political priorities.

The paper is organized as follows: Section 2 provides a description of the context of interactive robotics and their potential and actual adoption in different sectors. Section 3 describes the hypotheses and methodology of the study presented in the paper. Section 4 presents the main results on the analysis of the potential adoption of existing robotic systems in healthcare related contexts. Section 5 provides a discussion on the obtained results, the limitation of the provided study and some concluding remarks and guidelines.

## 2. Contextualization of interactive robots

Eurostat shows that a quarter of European industry uses robots in its operations, more in larger companies and less in small and medium-sized enterprises (18). Enterprises more commonly use industrial robots than service robots, moreover enterprises use service robots mainly for warehouse management systems (44 %) followed by transportation of people and goods (22 %), cleaning or waste disposal tasks, as well as assembly works (21 % each). The International Federation of Robotics (IFA) and the Robotics Industries Association (RIA) estimates that industrial robots will be a crucial part of the progress of the manufacturing industry for the foreseeable future (19, 20).

A worldwide comparison shows that in China sales volumes are measured categorized in three types: domestic service robots, medical service robots and public service robots. The statistics show that domestic service robots, including robotic tools and educational robots amount to 62% of the sales volume; medical service robots including robotic surgery, rehabilitation robots, auxiliary service robots and medical logistic robot amount to 24%; and public service robots including reception and guide robots, delivery robots and smart security robots amount to 14% of the sales volume (21, 22). The application of home service robots represented by sweeping robots is relatively mature; Public service robots have been used in retail, catering, government affairs, finance, hospitals, and other scenarios, but they have not yet shown an uptake in a large scale; there are high technical barriers in medical service robots. Hence, medical service robot industry is still in its infancy (22).

For the US, official documents regarding the existence of robots in various sectors or strategies for developing social robots are not available for the last 10 years. On behalf of North America, researchers and companies in both the US and Canada are at the forefront of the development and publication of scientific articles on the subject. Taking part of global forecasts, the expectations, developed on national levels are high. Transparency Market Reserach, which refers to social robots as artificial intelligence (AI) systems that are developed to interact with humans and other robots, includes social robots for tutoring, telepresence, companionship, and customer engagement in their external information. India is particularly highlighted as a nation in strong growth, especially when it comes to the deployment of social robots for traffic management (23).

Speculations about the social uptake of robots are contextualized in various fields and in terms of automation of tasks (24–27). Questions arise about whether robots help to strengthen social relationships or de-socialize human relationships (28). The increasing rationalization and automation in industry is well-documented, as is the development of medical technology and everyone's life experiences of technological change (29–31), while household work is less recognized. There is early research available on the automation of housework in the 1930's (32), while the growing trends toward working from home that includes home healthcare services, aging in place and demographic changes, are relatively new research areas. One aspect discussed is the possible level of keeping humans in the loop or outside of the loop. Biomedical engineering does not necessarily keep the user in the loop and can adhere very well to a technological rationality. From Albrecht Dürer's attempt to apply mathematics to the proportions of the human body, to Phelps' invention of PET imaging technology, patients have been notably absent from being involved in the examinations of their state of health (31). The organization of patient contacts managed by doctors and nurses does not always involve patients in medical procedures. This may change with the digitalization of healthcare, especially home help services, since these systems, including robots and artificial intelligence, and long-established technologies—such as telephones and alarms—require active participation by both medical professions and patients.

A model visualizing the degree of automation was published by Schraft and his colleagues in 1993, showing the differentiation between industrial and service robots (33). They suggest that automation is highest in industry, where there are predetermined tasks and an adjusted environment for automatic task execution. Service robots require a greater flexibility in the performance of various tasks. Personal robots involve communication with the environment and surveillance of available actions. The model demonstrates good compatibility with later calculations of automation potential. These calculations show that jobs in industrial production, service, sales, administration, and transport have a high probability of automation (70–100% of the work tasks) while jobs in education, care and health have a low probability of automation (0–30% of the work tasks) (34–36). Lack of understanding of contextual differences between industry and healthcare means that the introduction of new working methods with robotic support has a negative effect on work (37). The organization of home healthcare services based on the same predictability as in an assembly line has been shown to contribute to an impoverishment of the work content and increased control of workers as being merely part of the machine—a “digital Taylorism,” i.e., work is divided and controlled without scope for the flexibility required for the tasks. Such a lack of awareness in the implementation of robots in the care sector creates a contradiction between a technical rationality and a care rationality (38, 39).

Thus, there are contextual differences between different sectors that affect acceptance, and thus social uptake, of new technology. There are also some similarities. In all contexts where people are present, domestication of the technology occurs even if the concept borrows its context from domestication taken place in the home (40). Both in industry and in the home, there is a habit and experience of using technology, which affects the encounter with new applications. In both environments, there are both objects and social relationships, although the latter is more characteristic in-home care settings in line with Schraft's model.

The Technology Acceptance Model (TAM). developed at the end of the 1980's, models a person's acceptance and adoption of a technology according to perceived usefulness and perceived ease of use (41). The model originates from information system research, and mainly illustrates that a person's beliefs about the consequences of using a particular system will affect his/her attitude and in turn his/her actual behavior. This has been widely applied in working life. After being criticized for overlooking social and cultural factors, the model was expanded in 2003 into a unified theory (UTAUT) merging eight previously published acceptance models (41). Recent studies on the acceptance of robots in new fields of work confirm context-dependency (42). Beyond the acceptance of single applications in a defined social context, domesticated technology can, according to Rogers' theory, as described above, reduce uncertainty regarding new applications if the former and the latter resemble each other. Completely new applications, no matter how well-developed, may be rejected if the potential adopter cannot relate to the new way of doing things.

However, other studies show that familiarity is not always the most relevant approach to adoption. A comparison of organism-based and object-based approaches shows that robot design plays an essential role in the consumer's acceptance of robots. While organism-based robots are designed to imitate behaviors or simulate emotions—such as Kismet, a robot head made in the late 1990's as an experiment in affective computing—object-based designs are robotising everyday objects such as vacuum cleaners. Users accept object-based robots before organism-based robots, even though the latter was perceived as more familiar. Following the design principle “form follows function,” the explanation given is that the product is defined by its delivery of a specific function, not necessarily by it imitating the user (10).

## 3. Methods

This study assesses the adoption potential of interactive robots occupying the upper levels of the Technology Readiness Level (TRL) scale. TRL is a scale used to estimate technology readiness. This scale, invented by NASA in the 1970s, became a standard (ISO 16290:2013) and was recommended by the European Commission to be used in the Horizon 2020 programme (1). The TRL consists of nine stages varying from 0 (the preliminary idea) to 9 (full commercial solution) and is summarized in Table 2 for the sake of completeness.

### 3.1. Participants

The stakeholders that participated in this study include 14 universities and research labs, six businesses and five authorities or consultants including a broad competence shown in Table 1. The businesses include engineering, design, and testing of interactive robots, rehabilitation, clinical applications for neurological disorders, haptics, exoskeletons, smart systems, materials and road systems, infrastructure, industrial environments, aerospace, education, ethics, and law. Universities and research labs cover the areas of robot engineering, user-centered design, affordable design, social human-robot interaction, cognitive science, and society and technology studies. The facilitators are authorities and consulting firms promoting the deployment of robots, as well as commercialization, standardization, and regulatory frameworks.

The data was collected over 9 months with an instrument described below and developed specifically for this purpose. A description of selected examples of technologies is presented. This selection is limited to commercial applications and systems, technology available to consumers, and technology provided to users within the framework of social welfare systems. These stakeholders, by virtue of their knowledge of the existence of examples of interactive robots, collected the data and submitted their evaluations of each example.

The total number of people involved in the sample is 25, the same as the number of institutions involved. This can be considered a convenience sample, as the participants are all partners in the Horizon 2020 project INBOTS. They were assessed by the European Commission as a representative sample to identify gaps and needs related to the current level of understanding of robotics among the public, outside of industry; and to address the lack of a clear understanding and communication between research, business, and society. Project participants were representative components of potential stakeholders that collaborated in the project to identify the most important aspects needed for an effective responsible research and innovation in robotics. As previously introduced, INBOTS structure covered the technical expertise pillar, the business expertise pillar, the ethical, legal, and socio-economic (ELSE) expertise pillar, as well as the end-users, policy makers and general public pillar.

Furthermore, each stakeholder could involve additional staff in the work of this study within their own organization. The selection of stakeholders is limited by the fact that it is not a representative sample in the sense of being a random selection of subjects. Despite this, a unique breadth of competencies has assessed the technology readiness and social uptake of robots in the health field.

### 3.2. Description of technologies

Several robot applications dedicated to the health field were collected by the stakeholders and delivered to the coordinator of this study, who made a first analysis based on the developed evaluation instrument. The coordinator's assessment was then reviewed by the entire project consortium. More specifically, to get as close as possible to social uptake and avoid applications that are still at the prototype stage or are far from potential users and consumers, an inclusion criterion was that the best practices should be high on the Technology Readiness Level. Furthermore, very specialized and specific solutions, that cannot be considered potentially useful for a significant percentage of the society, were excluded from the analysis. The dataset of the collected robots was composed of 75 examples. For each analyzed solution, the following information was collected:

- Name.
- Short description.
- Company/project.
- Country.
- Link to an external resource (webpage, video, paper, etc.).
- Type of robot.

**Figure 1:**
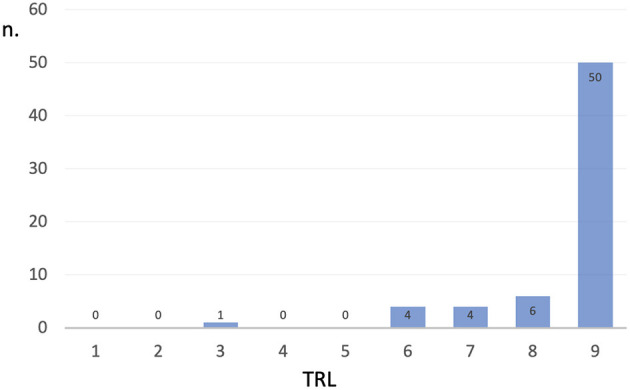
Distribution of the TRL values for the 75 robots collected in the analysis.

**Figure 2:**
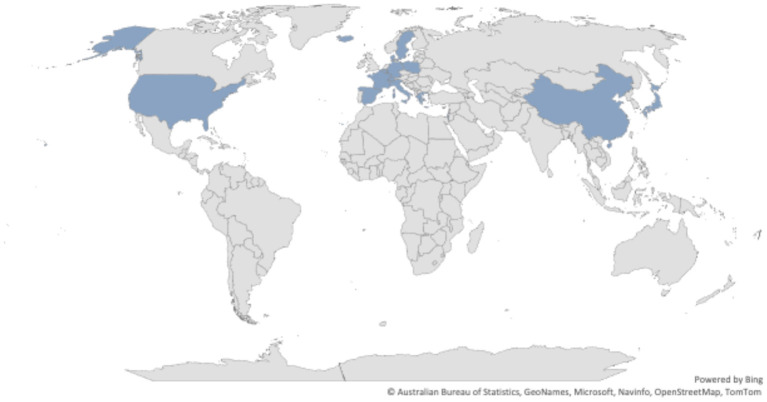
Distribution of the countries for the 75 robots collected in the analysis.

**Figure 3:**
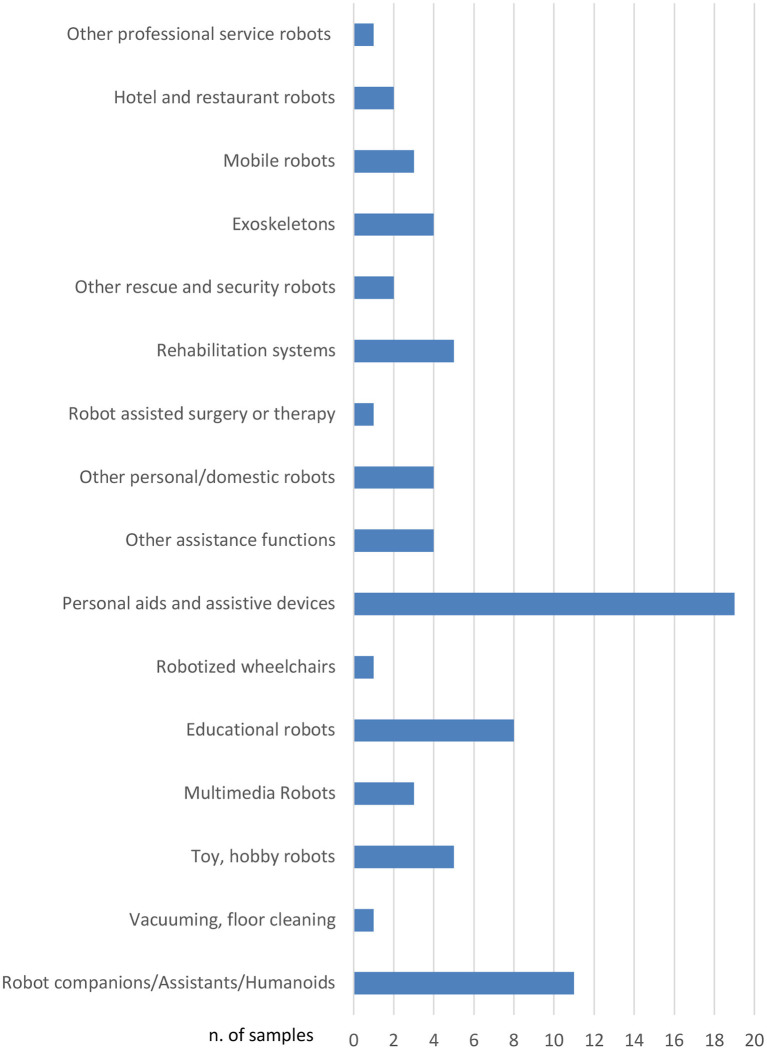
Type of robots distribution for the 75 robots collected in the analysis, according to the classification proposed in Turja et al. (15) and summarized in Table 3.

For what concerns the type of robot, we referred to the taxonomy proposed by the International Federation of Robotics (IFR) detailed in the World Robotics—Service Robots report for 2018 and 2019 and summarized (43) and reported in Table 3. Figure 1 represents the distribution of TRL values in the dataset of collected robots, it can be noticed that mainly robotic systems with high TRL values have been included. Figure 2 shows the distribution over the different countries of the companies and institutions providing the collected robots, while Figure 3 shows the distribution of robot types according to the taxonomy reported in Turja et al. (15).

**Table 3:** Service robot classification according to IFR-UNECE (43).

1–7 Robots for domestic tasks	30-33 Construction and demolition
1-Robot companions / assistants / humanoids	30-Nuclear demolition & dismantling
2-Vacuuming, floor cleaning	31-Building construction
3-Lawn mowing	32-Robots for heavy /civil construction
4-Pool cleaning	33-Other construction and demolition systems
5-Window cleaning	**34-38 Logistic systems**
6-Home security & surveillance	34- Autonomous guided (AGV) vehicles in manufacturing environments
7-Others	35-AGVs in non-manufacturing environments (indoor)
**8-11 Entertainment robots**	36-Cargo handling, outdoor logistics
8-Toy/hobby robots	37-Personal transportation (AGV for persons)
9-Multimedia robots	38-Other logistics
10-Education and research	**39-42 Medical robotics**
11-Others	39-Diagnostic systems
**12-14 Elderly and handicap assistance**	40-Robot assisted surgery or therapy
12-Robotized wheelchairs	41-Rehabilitation systems
13-Personal aids and assistive devices	42-Other medical robots
14-Other assistance functions	**43-45 Rescue und security applications**
15-Other personal/domestic robots	43-Fire and disaster fighting robots
**16-21 Field robotics**	44-Surveillance/security robots without UAV
16-Agriculture (broad acre, greenhouse, fruit-growing, vineyard)	45-Other rescue and security robots
17-Milking robots	**46-50 Defense applications**
18-other robots for livestock farming	46-Demining robots
19-Mining robots	47-Unmanned aerial vehicles
20-Space robots	48-Unmanned ground-based vehicles (e.g., bomb fighting)
21-Others	49-Unmanned underwater vehicles
**22–26 Professional cleaning**	50-Other defense applications
22-Floor cleaning, professional	51 Underwater systems (civil/general use)
23-Window and wall cleaning (including wall climbing robots)	52 Powered Human Exoskeletons
24-Tank, tube and pipe cleaning	53 Mobile Platforms (general use)
25-Hull cleaning (aircraft, vehicles, etc.)	54–58 Public relation robots and joy rides
26-other cleaning tasks	54 Hotel and restaurant robots
**27–29 Inspection and maintenance systems**	55 Mobile guidance, information, telepresence robots
27-Facilities, plants	56 Robots in marketing
28-Tank, tubes, pipes and sewers	57 Robot joy rides
29-Other inspection and maintenance systems	58 other public relation
	59 Other professional service robots not specified above

**Figure 4:**
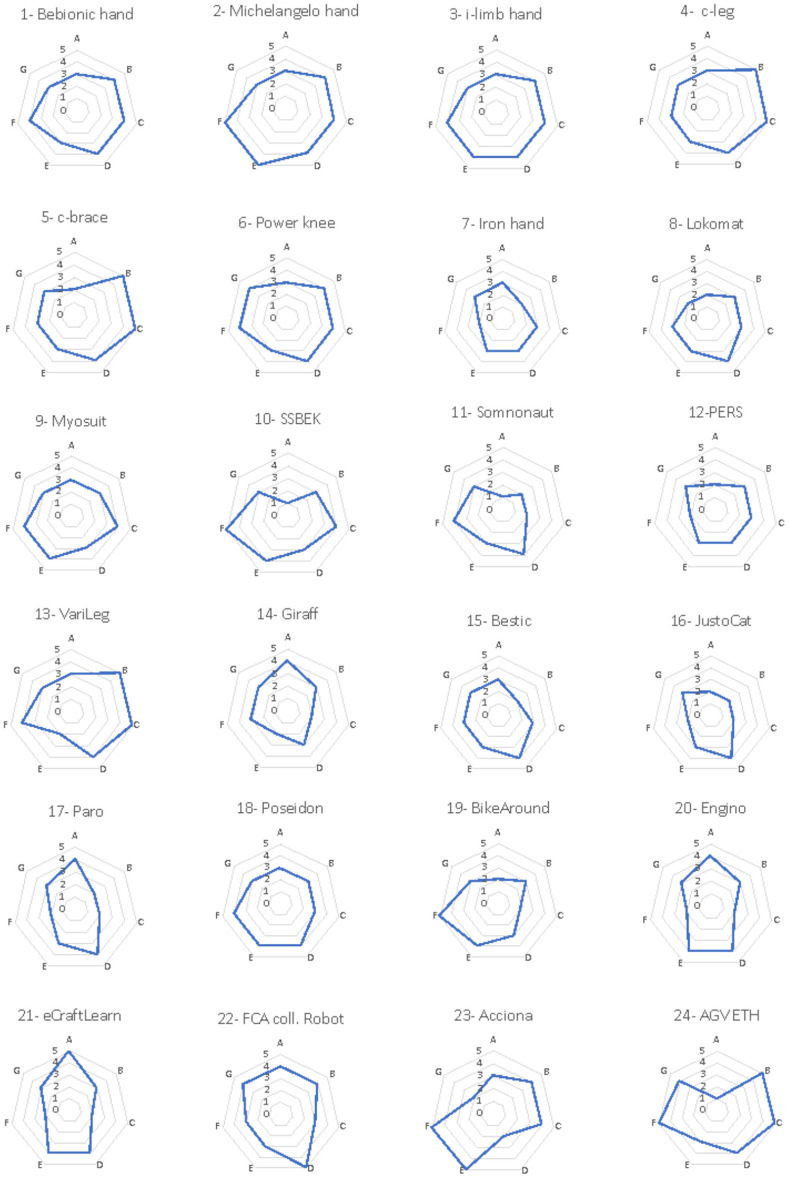
Evaluation of the analyzed robotic solutions. Seven criteria were used to evaluate each of the analyzed solutions, namely, A-demand, B-usefulness, C-relative advantage, D-feasibility, E-attractiveness, F-novelty, G-level of confidence. For each criterion, an evaluation grade ranging from 1 to 5 was assigned, the obtained results were represented by a spider-net diagram.

A representative subset composed of 24 systems has been considered in this paper for the sake of conciseness (see Table 4 and Figure 4).^2^ Table 4 provides a more detailed description of the robot applications included in the evaluation. They are all defined as interactive robotic applications or systems being tested in intended environments on their way to being implemented and commercialized, or already commercialized and available to consumers or users.

**Table 4:** Description of robot applications included in the evaluation.

**#id**	**Name**	**Description**	**Company/ project**	**link**
1	Bebionic Hand	Multi-articulating myoelectric hand with several selectable grip patterns and hand positions enable the user to perform everyday activities.	Ottobock	https://www.ottobock.com/en-gb/product/8E70
2	Michelangelo Hand	Robotic hand prosthesis. to feature an electronically actuated thumb which mimics natural human hand movements. Owned by Otto Bock.	Ottobock	https://www.ottobock.com/en-gb/product/8E500
3.	i-limb hand	Bionic hand, a biologically inspired prosthetisis, with individually powered digits and thumb and a choice of grips. Offers full hand solutions in addition to partial hand solutions.	Ossur	https://www.ossur.com/en-gb/prosthetics/arms/i-limb-access
4	C-Leg.	Prosthetic knee joint controlled by a microprocessor interacting with the user.	Ottobock	https://www.ottobockus.com/prosthetics/lower-limb-prosthetics/solution-overview/c-leg-above-knee-system/
5	C-Brace	Prosthetic leg with microprocessor sensor technology enabling flexible mobility while sitting down, navigating slopes, walking on uneven terrain, or going downstairs.	Ottobock	https://www.ottobockus.com/orthotics/solution-overview/c-brace/index.html
6	Power Knee Prosthetic	Motor powered knee joint, providing active motion and stability to replace lost muscle function.	Ossur	https://www.ossur.com/en-us/prosthetics/explore-power-knee
7	Ironhand	Robotic muscle strengthening system for professional use to reduce fatigue and injury due to the repetition of the same grip motion.	Bioservo Technologies	https://www.bioservo.com/professional
8	Lokomat	Gait training tool for rehabilitation.	Hocoma	https://www.hocoma.com/solutions/lokomat/
9	Myosuit	Wearable robotic system (exoskeleton) supporting patients with muscle weakness in hip, knees and ankles during rehabilitation and physiotherapy training	MyoSwiss	https://myo.swiss/en/
10	SSBTEK (Social Welfare Services)	Digital service-AI-for social welfare. SSBTEK is a service for citizens' applications for financial assistance and enables municipalities.	The Swedish Association of Local Authorities and Regions.	https://skr.se/skr/integrationsocialomsorg/ekonomisktbistandforsorjning/automatiseringekonomisktbistand/ssbtekdigitaltjanstforekonomisktbistand.2998.html
11	Somnomat	Autonomous robotic platform able to monitor the user during sleep and to interact with him to improve sleep quality.	Sensory-Motor Systems Laboratory at ETH Zurich.	https://sms.hest.ethz.ch/research/current-research-projects/somnomat.html
12	PERS	Personal emergency response systems, used in nursing homes and patients own homes.	-	-
13	VariLeg	Assistive device for lower limbs, like exoskeletons, to restore natural gait for persons with complete loss of motor functions in their legs.	Rehabilitation Engineering Laboratory at ETH Zurich.	https://www.varileg-enhanced.ch/
14	Giraff.	Care robot used for virtual visits in the homes of care receivers and possible for care givers to control remotely.	Camanio	https://www.camanio.com/en/welfare-technology/
15	Bestic	Robotic eating tool for people with impaired function, or have no function in the arms or hands.	Camanio	https://www.camanio.com/en/welfare-technology/
16	JustoCat	Robotic animal shaped as a cat used as a supplement to care or as a company for people with dementia or intellectual disabilities.	Camanio	https://www.youtube.com/watch?v=Zt0pEWD_pmQ
17	Paro	Therapeutical robot-animal shaped as a seal and used as a supplement to care or as a company for people with dementia or intellectual disabilities.	Parorobots	http://www.parorobots.com/
18	Poseidon	Hygiene robot that helps the elderly and disabled to independently manage their personal hygiene.	Robotics Care AB	http://roboticscare.com/
19	BikeAround	Digital bicycling tool with a control and tram unit and software that, with the help of Google Street view, makes it possible to experience places all over the world.	Camanio	https://www.camanio.com/en/welfare-technology/
20	Engino robotics	Robotic system designed for primary and secondary level students enabling intellectual developments as an upward expanding spiral in which children must constantly reconstruct the ideas formed at earlier levels with new, higher order concepts acquired at the next level.	Engino robotics	https://www.enginorobotics.com/
21	eCraft2Learn	Unified User Interface developed in the eCraft2Learn project aiming at providing tools and a learning methodology enabling teachers and students to make their own robots, using open source innovative technologies, low cost or recycled materials, digital fabrication and the DIY philosophy.	eCraft2Learn project	https://project.ecraft2learn.eu/
22	Universal robots' collaborative robots	Collaborative robots increasing ergonomics and efficiency of the workstation in Fiat Chrysler Automobiles (FCA) including for example the use of exoskeletons at workstations difficult to automate due to high flexibility of the process and the need of the humans' experience.	Universal robots	https://www.universal-robots.com/
23	Wheeled robots	Wheeled robots are used logistics for carrying parcels. For example, wheeled robots with autonomous navigation can be used for logistic tasks in work sites to avoid accidents and extreme weather conditions for workers.	-	-
24	Automated guided vehicles (AGV)	AGV can be used in hospitals being part of the logistics, to pick up carts, transport clothes and stock up on medical supplies.	-	-

The interactive characteristic of the robots refers to the user in the loop, activating or controlling the robot. This excludes systems monitoring human behavior without any direct interaction between the robot and the user. Solutions that did not refer to a specific prototype or product in the early stages of testing were excluded from the study. The conditions for carrying out the European Commission project also excluded military solutions and sex robots from the study.

The set of examples includes products that can be evaluated as either a pre-commercialized prototype; a commercial system already launched; or as a full commercial application available to consumers. Education-oriented solutions are also included because education in (and with) robotics, particularly if it incorporates the making culture (“make your own robots”), can play an important role in fostering improved understanding of, familiarization with, and acceptance of robots, and contribute to the development of a future robotics society (13). More specifically, robotics education can help address features of Rogers' theory of diffusion of innovation, such as complexity (robots are perceived as relatively difficult to understand and use) and trialability (robots may be tried and modified).

### 3.3. Evaluation instrument and analysis

The evaluation of adoption potential inspired by Rogers' theory of diffusion of innovation (2) and design principles of attractiveness was based on seven parameters. The evaluators were asked to give a score out of five for each of these parameters. The scores ranged from 1 (not in demand, not useful, no relative advantage, not feasible, not attractive, no novelty) to 5 (strongly in demand, extremely useful, excellent relative advantage, and extremely feasible, extremely attractive, high novelty). The participants answered to the following questions for each of the analyzed solutions:

A. **Demand** How big is the market share? How many estimated users might need the solution?
B. **Useful/usability** How useful and easy to use is the solution? Can the users use it by them self or would they extensive need training or assistance in order to be able to use the solution?
C. **Relative advantage** The degree to which an innovation is perceived as better than the idea it supersedes by a particular group of users in terms that matter to those users, like economic advantage, social prestige, convenience, or satisfaction.
D. **Feasibility** How feasibly is it to put this into practice? It may have been a really attractive solution to use a time machine, but is it really feasible?
E. **Attractiveness** How attractive is this as a solution? What is the appeal of the solution? Does it completely solve the problem? Or is it only a partial solution?
F. **Novelty** How novel is the idea? If it isn't novel for this situation, it probably isn't very creative
G. **Level of confidence** How confident to do feel about your marking?

## 4. Result

### 4.1. Description of the technologies

The subset of robot examples collected by the stakeholders are described in detail in Table 4.

Most of the examples are dedicated to individual rehabilitation or frailty and stress. Robot applications for individual rehabilitation include prostheses/exoskeletons, mainly for hands (no. 1, 2, 3, 4, 5, 6, 7, 8, 9, 13) and legs (no. 4, 5, 6, 8, 9, 13). This include Myosuit which is an exoskeleton, a wearable robotic system supporting patients with muscle weakness in hip, knees and ankles during rehabilitation and physiotherapy training (no. 13).

Another type of exoskeleton used at workstations difficult to automate is the Collaborative robot at Fiat Chrysler Automobiles (FCA, no. 22). This exoskeleton is used to increase both ergonomics and efficiency at workstations.

Another robot application that is a bit different from prosthesis and used to replace hands is Bestic. This is a robotic eating tool for people with impaired function or no function in arms and hands (no. 15). Poseidon is another application with a focus on the whole body, a shower robot that can assist people with various forms of mobility impairment to take care of their personal hygiene (no. 18).

The robot applications focusing on frailty and stress were designed as stuffed animals, a seal and a cat (no. 16, 17). The target group for these examples is mainly elderly care. This also applies to the Giraff, a care robot to be used for virtual visits in the homes of care receivers and possible for care givers to control remotely (no. 14). Another example of counteracting stress is the Somnomat, an autonomous robotic platform able to monitor the user during sleep and to interact with him or her to improve sleep quality (no.11).

The Giraff is an example of a robot application for people who are dependent on others for help or service (no. 14). One system for service in a broader sense identified by the stakeholders is Social Welfare Services (SSBTEK, no. 10). This is a digital service for citizens applying for social welfare. This application is on the verge of how interactive robots are defined in this study but has nevertheless been included since individual users are interacting with the system. For the moment it is owned by The Swedish Association of Local Authorities and Regions.

Other examples included in the evaluation are personal emergency response systems used (no. 12). This type of alarm system has been around for a long time and is now digitalized with new functions that can be connected to care robots and interact in different ways with the user. Another application taking the user into virtual realities is BikeAround (no. 19). This is a type of robot application that aims to stimulate people with problems to move around by themselves. It is a a digital bicycling tool with a control and tram unit and software that, with the help of Google Street view, makes it possible to experience places all over the world.

Another type of robot that also aims to stimulate through learning and personal growth are the Engino robotics and the concept eCraft2Learn (no. 20, 21). Engino robotics is designed for Primary and Secondary level students enabling intellectual developments as an upward expanding spiral in which children must constantly reconstruct the ideas formed at earlier levels with new, higher order concepts acquired at the next level. eCraft2Learn is a Unified User Interface developed in the eCraft2Learn project aiming at providing tools and a learning methodology enabling teachers and students to make their own robots, using open-source innovative technologies, low cost or recycled materials, digital fabrication and the Do It Yourself (DIY) philosophy.

Logistics and collaborative robots for hospitals are also a development trend, here exemplified by Wheeled robots and Automated guided vehicles (AGV) moving around in human environments interacting with people (no. 23, 24). Wheeled robots with autonomous navigation for logistic tasks in work sites are carrying parcels to avoid accidents. Automated guided vehicles (AGV) are robots used in hospitals to pick up carts, transport clothes and stock up on medical supplies.

### 4.2. Technology readiness

Figure 1 summarizes the technology readiness of the solutions considered, it can be observed that most of the analyzed solutions are at the highest level of the TRL scale, which is Level 9 (Full commercial application available to consumers). In the analyzed subset, five solutions did not reach the highest level even though they were proposed by the stakeholders as technology-ready interactive robots. These are the unified user interface eCraft2Learn (no. 21) and wheeled robots used in logistics for carrying parcels (no. 23 and 24), both of which reach Level 6 (Prototype system tested in intended environment); the exoskeleton VariLeg (no. 13) which reaches Level 7 (Pre-commercial demonstration system), and the collaborative robot (no. 22) which reaches Level 8 (Commercial system launched). The wheeled robot—a high-tech experimental robot that drives autonomously and decides which path to take in a completely unstructured and chaotic environment such as a worksite—is considered as an interactive application in hospitals; and in fact, the AGV robot (no. 24) is already installed and used at the new Karolinska Hospital in Sweden, collaborating with nurses by carrying and sorting equipment in storage areas.

### 4.3. Adoption potential

**Figure 5:**
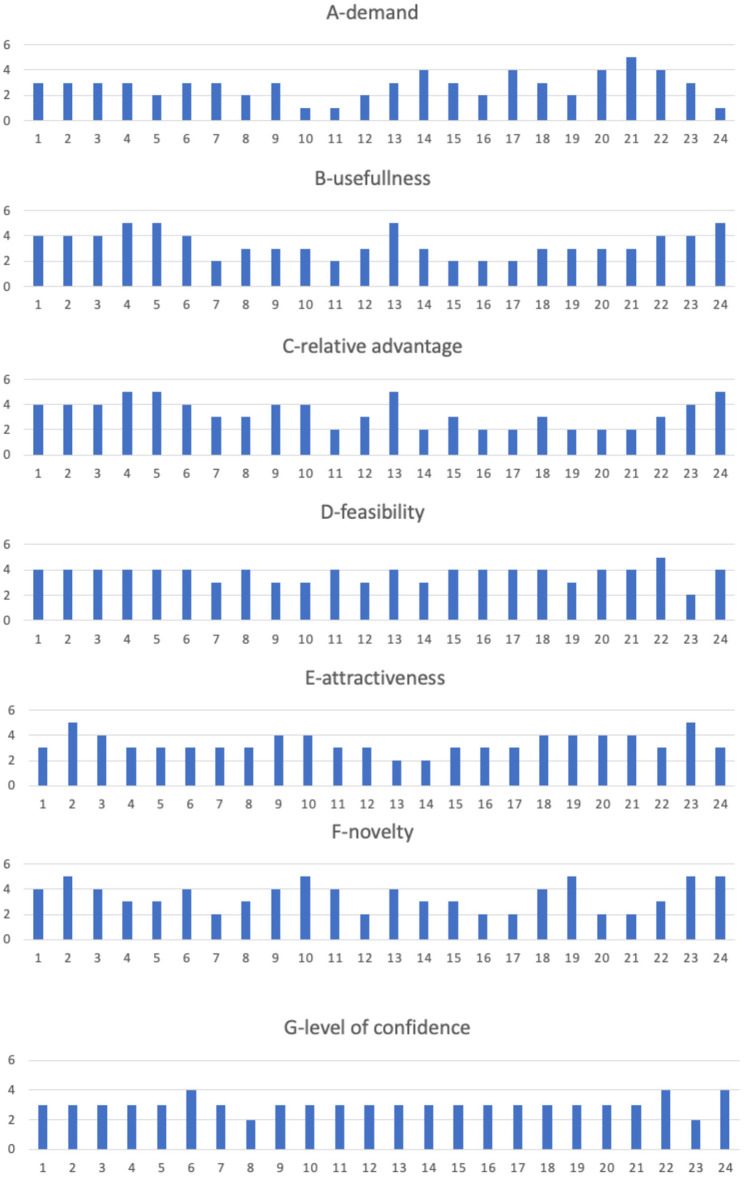
Evaluation of the analyzed robotic solutions. Seven criteria were used to evaluate each of the analyzed solutions, namely, A-demand, B-usefulness, C-relative advantage, D-feasibility, E-attractiveness, F-novelty, G-level of confidence. Each diagram compares, for each criterion, the evaluations collected for the 24 analyzed solutions.

The results are represented in spider-web diagrams (Figure 4) and histograms (Figure 5) showing the value of each of the evaluation criteria: A-demand, B-usefulness, C-relative advantage, D-feasibility, E-attractiveness, F-novelty, G-level of confidence. For each category, the possible score ranges from 1 (very low) to 5 (very high). In particular, to highlight the evaluation of each robotic solution, Figure 2 summarizes the evaluation of the adoption potential for the 24 robotic solutions according to the seven evaluation criteria, while Figure 3 reports, for each of the seven mentioned criteria, the results obtained by the analyzed system.

Demand (A) was scored low for the majority of all the listed solutions except for the educational solutions and the collaborative robot aiming at improving both ergonomics and efficiency at workstations: Engino, eCraft2Learn and FCA (no. 20, 21, 22). The fact that the demand criterion received low scores in most examples is not surprising since only a few have been implemented and have been subjected to some form of valuation by consumers or have been procured. The robots that received high scores have been tested and evaluated in practice. Thus, it may be easier to determine whether there is a demand or not.

On the other hand, scores for usefulness (B) were high for all the solutions except for the Iron hand, Somnomat, JustoCat, and Paro (no. 7, 11, 16, 17). That the criterion usefulness, being close to usability, received high scores in most examples is also not surprising since this criterion is tested on each development level on the TRL scale and hopefully usability increases the higher up on the TRL scale an application is. However, three examples got low usefulness scores. Three of them being applications to counteract stress and worries, the Somnomat, JustoCat and Paro, might lack such tests.

Relative advantage (C) was scored high, especially for those that enable people to walk, grip, or carry out other actions that were not possible before. For other solutions, this parameter is highly dependent on the context and dependent on what was there before or other available alternatives for carrying out different tasks. Two such solutions were given lower scores—Somnomat and Giraff (no. 11, 14). This difference whether the application is dependent on the environment or not can be an explanation for the difference in points.

Feasibility (D) was evaluated as moderate as or higher than moderate for all solutions except for wheeled robots in logistics for carrying parcels (no. 23). The latter solution also dependent on the environment, might be challenged by a harsh and demanding environment.

The scores for attractiveness (E), i.e., how well the solution meets needs or solves problems, were the most varied, ranging from 2 to 5. The question of attractiveness is not only a question of function but perhaps above all of design and of the user's taste. The results clearly show that stakeholders found that this is a criterion that must be further developed for these examples to be adopted.

Novelty (F) was generally scored high, except for some of the prosthetics, and was low for the learning solutions (no. 4, 5, 7, 8, 20, 21). Only the c-brace and Paro were scored moderate by the stakeholders. That these examples have a news value goes without saying.

The criterion confidence (G) has been included in the evaluation as a way of measuring stakeholders' confidence in the possibility that these examples have the potential to be adopted.

Following the criteria of what affects the adoption potential the result shows that these examples are expected to have a value as useful, new and have a relative advantage over previous solutions. In terms of feasibility, there is a difference between applications that are systemic and dependent on functioning in a social environment compared to more limited functions. More uncertain is which design can make the applications more attractive.

## 5. Discussion

### 5.1. Comments on the obtained results

This study contributes to enhancing the discussion of the benefits of interactive robots in promoting health. The results indicate the need for further investigation about the connections between technology readiness, and adoption and use. Notwithstanding the limitations discussed in the following, the results of this study show that to be able to assess the adoption potential, more knowledge is required about the demand, the users' requirements for attractive applications and the necessary consideration for the environment.

The results suggest that concerning the usefulness of robots in the healthcare, it is not possible to discuss the benefits of interactive robots based only on trends and challenges. This study confirms that the adoption potential of a technology-ready solution cannot be evaluated independently from the social context in which it is intended to be used. This raises questions about how the usefulness of these robots is evaluated and to what extent users and consumers can influence the development, design, and evaluation of new robot applications: do sufficient methods even exist to explore needs and requirements in contexts not yet recognized for robotic developments?

Most of the robot applications included in this study own some form of novelty, but the application areas are still traditional as they are based on health defined in terms of medical needs and disabilities. New approaches to health, such as wellness and lifelong learning, can broaden the perspectives of what constitutes health and gives the individual increased opportunities to take responsibility for their health. This includes both personal growth, learning and somatic design bringing added value to caring for one's own body. Care robots and those for sleep support may have the potential to meet these needs. To find out, participatory design and involvement in innovation processes is key.

In this respect, technology reflects the power and configurations of users and user contexts, or as Latour says: “Technology is society made durable” (43, 44). Considering the differences in attitudes, values and ideologies between European countries, not least variations in the organization of welfare and healthcare, the examples reported in this paper might have very different routes to closure. In Sweden, for example, it is reported that the government's investment in digitalization is progressing much more slowly than expected (45). For someone to get a prosthetic or orthotic device, for example, they need to go through insurance, otherwise it is too expensive for them. The insurance terms can vary greatly between countries and set limits on what they cover for each patient, thus limiting the early adoption of novel technologies (46).

Regarding the education-oriented solutions, especially those incorporating the making culture (“make your own robots”), future research should focus on longitudinal surveys that examine the long-term impact of these solutions on the younger generation in terms of the demystification of, familiarization with, and acceptance of robots. Such a research direction would also help designers of educational robots to adapt their solutions and products according to the results of the surveys incorporating feedback from educators and learners.

Speeding up or slowing down the European development of robots depend on support for social development. In Europe this is not as strong as the support for industrial development. Lack of access to statistics about social uptake and acceptance of robots Europe-wide is a problem that tends to lower development speed and target fulfillment. Without statistics on the social uptake and use of digital applications, at the start of what seems to be a new technological wave of interactive robotics, policymakers are flying blind (47).

Another factor affecting acceptance of robotic applications in Europe are regulations at EU level. Europe can be seen as one of several sociocultural ways of treating digitalisation that is an alternative to the dominant US American model (free markets, neo liberalism, “surveillance capitalism” dominated by few global players) (48) or the emerging totalitarian paradigm of surveillance in China. In the European context, the factual acceptance of robots depends on normative values of acceptability that are represented in EU regulations such as the “Ethics Guidelines for Trustworthy AI” prepared by the High-Level Expert Group (HLEG) on AI. Here, values such as transparency, respect for privacy and ecological sustainability are assigned an important influence on the social context of robotic applications in Europe. For Europe, the diversity of the healthcare systems and welfare systems that could procure robots will continue to be an obstacle if a common policy is not agreed. eCraft2Learn is an example that shows promising results but needs to be procured by public authorities or schools in municipalities. This also points to the fact that the TRL scale used in the analysis of the examples collected in this document is not perfect for understanding procurement. A model that also includes social robots that meets the needs of public demand and that corresponds to public procurement is important for technological development to be utilized outside industry.

To sum up, the evaluations presented in this work confirm the assumption that robots are not yet adopted despite their high technical maturity. Design and contextualization are aspects that need to be considered for social uptake. More instruments that not only measure innovations in relation to markets but also consider procurement and public goods need to be developed. The use of robots in industry still dominates statistics and theories about what influences the adoption of new technology.

### 5.2. Limitations

It's worth to underline that this study is not based on a randomized selection of examples: the solutions were selected on the basis of desk research to provide a comprehensive overview of the available resources in robotic solutions for healthcare, their development stage and their main applications.

A further limitation of the study is the lack of users as participants. Out of the 25 stakeholders, none is clearly a potential user, so the convenience sample could present a bias. However, as discussed in the introduction, the stakeholders involved in the analysis are participating to a more general discussions on the role of interactive robots in the society from different multidisciplinary points of view. We acknowledge that using as participants directly project partners could represent a limit in the study, but at the same time they could bring in the study the experience, the discussions and the results from their other complementary activities.

In this paper the criteria adopted to evaluate and compare robotic solutions are based on Rogers' Theory of Diffusion, TAM and UTAUT, it's worth to observe that none of these methodologies consider service-related aspects, which impact in the adoption (49). Furthermore, the TRL scale is limited to the extent that it does not consider the implementation in real life. Admittedly, the TRL scale includes levels for large-scale prototype testing in intended environments and pre-commercial demonstration systems.

### 5.3. Concluding remarks

The use of robots has reached different stages of maturity in different industrial sectors. Available statistics and best practices show that industries that are at the leading edge include manufacturing, logistics, rescue activities and exoskeletons. Promising areas with a number of applications implemented include support for disabilities, robotized assistance for surgery and education.

Robots in hospital environments are increasingly common today both for surgery and for transportation: robots for surgery are commercially available and well established in all surgical subspecialities allowing laprascopic surgery instead of open surgery, as for instance the da Vinci robot. Robots defined as service robots being part of hospital logistics are used for cleaning and deliveries, for example for picking up carts, transport clothes and stock up on medical supplies.

Education is another research area expected to grow. Though the industry of educational technologies has already provided a lot of educational robots for formal and informal learning, as highlighted also from the above-described review, a new pedagogical trend, inspired from the maker movement, has recently emerged promoting the incorporation of the making culture in robotics education. This new paradigm (that we could summarize with the motto “make your own robot”) is expected to give a boost to Do-It-Yourself robots and reduce the demand for ready-made robots. Ongoing research could benefit from longitudinal surveys to examine the long-term impact of the different education solutions on young generation in terms of demystification, familiarization with and acceptance of robots (13). Such a research direction would forthcoming help designers of educational robots to adjust their solutions and products according to the results of the surveys including feedback from educators and learners.

In social welfare, robots are used for administrative matters to support citizens' applications for welfare services, for example the SSBTEK owned by The Swedish Association of Local Authorities and Regions. Service robots, sometimes called care robots or social robots, are also provided to patients in care facilities to decrease anxiety, designed as animals, for example Paro or JustoCat (50–53). Robots able to interact with patients are on the way to be implemented in ordinary homes, as the shower robot Poseidon provided by Robotics Care Inc. There is also a range of adaptive robot hands and tele robots, for example the Giraffe robot, but the procurement of these products is still low.

The social uptake of robots is at the beginning of its development toward a wider use among the public. To promote this development requires:

- *Create and update more thorough knowledge about potential users and areas of use*. Knowledge is important in order to make necessary priorities. Today there is a lack of knowledge about areas of use and potential users beyond manufacturing and beyond rhetoric and “wish to be needs.” Statistics on uptake, sales volumes and use of robots in society beyond industrial sectors are needed. These statistics should include citizens, meaning all ages, with no upper age limit since the older citizens constitute the fastest growing age groups in the population.
- *Go beyond consumer markets*. Conditions for uptake of robots in promising areas such as education, disabilities and health are very different from individual consumer markets and from using robots in manufacturing. It is a matter of procurement of public goods and welfare services. To explore this potential, the awareness of the context of the use of robotic applications outside manufacturing and traditional working life aspects, needs to be prioritized among researchers and in industry.
- *Make use of policy making*. Policy labs is a resource in a number of European countries dedicated to developing policies for the sake of societal challenges in cooperation with relevant actors. Robotics is an available resource for meeting challenges such as climate change and demographics but it is also the opportunity for companies interested to broaden their markets. Policies to increase the utilization of robotic resources should put change in focus, not separate technological applications. These changes must be based on the understanding of the system from within that is subject to change and include the people who populate the system.

## Data availability statement

The original contributions presented in the study are included in the article/supplementary material, further inquiries can be directed to the corresponding author.

## Author contributions

BÖ organized the work, coordinated data collection, and guided the analysis. BÖ and MM managed the manuscript writing. JM coordinated the project. All the authors contributed to the data collection and analysis and revised the paper.

## Footnotes

^1^ https://robots.ieee.org

^2^The overall dataset is available at the following link https://docs.google.com/spreadsheets/d/1ftJzUEEu-hcgIE_tKbyA3G48G7yFVli9/edit?usp=sharing&ouid=106564985096851388971&rtpof=true&sd=true.
